# Combined value of red blood cell distribution width and global registry of acute coronary events risk score on predicting long-term major adverse cardiac events in STEMI patients undergoing primary PCI

**DOI:** 10.18632/oncotarget.24128

**Published:** 2018-01-10

**Authors:** Xue-Wei Chang, Shou-Yan Zhang, Hao Wang, Ming-Ming Zhang, Wei-Feng Zheng, Hui-Fang Ma, Yun-Fei Gu, Jing-Han Wei, Chun-Guang Qiu

**Affiliations:** ^1^ Department of Cardiology, The First Affiliated Hospital of Zhengzhou University, Zhengzhou, Henan 450052, China; ^2^ Department of Cardiology, Luoyang Central Hospital Affiliated to Zhengzhou University, Luoyang, Henan 471009, China

**Keywords:** myocardial infarction, red cell distribution width, coronary artery, percutaneous coronary intervention

## Abstract

The combined value of RDW and GRACE risk score for cardiovascular prognosis in ST-segment elevation myocardial infarction (STEMI) undergoing primary percutaneous coronary intervention (PCI) has not been fully investigated. This study was designed to explore the combined value of RDW and GRACE risk score on predicting long-term major adverse cardiac event (Mace) in STEMI patients undergoing primary PCI. This study included 390 STEMI patients. The primary endpoint at the (33.5 ± 7.1) months follow-up was composed of cardiac death and nonfatal myocardial infarction. The relationship between clinical parameters and clinical outcomes was evaluated using Cox regression model and receiver operating characteristic (ROC) analysis. Mace occurred in 126 (32.3%) patients including 54 (13.8%) cardiac deaths and 72 (18.5%) nonfatal myocardial infarctions. Patients in Mace group had significantly higher RDW and GRACE score than the patients in non-Mace group. According to the Cox model, RDW and GRACE score were the most important independent predictors of Mace and cardiac death. The best cut-off value for RDW to predict the occurrence of primary events was 13.25% (AUC = 0.694, 95% CI:0.639–0.750, *P* < 0.001) and that for GRACE score was 119.5 (AUC = 0.721, 95% CI:0.666–0.777, *P* < 0.001). The combination of RDW and GRACE score were more valuable (AUC = 0.775, 95% CI: 0.727–0.824, *P* < 0.001). Kaplan–Meier analysis provided significant prognostic information with the highest risk for cardiac death (Log-Rank χ^2^ = 24.684, *P* < 0.001) in group with both high RDW (> 13.25%) and high GRACE score (> 119.5). The combination of RDW level and GRACE score may be valuable and simple independent predictors of Mace and cardiac death in STEMI patients undergoing primary PCI. They may be useful tools for risk stratification and may indicate long-term clinical outcomes.

## INTRODUCTION

Red blood cell distribution width (RDW) is a parameter of circulating erythrocytes measured by hematology analyzer. It is calculated automatically or manually by formula and expressed as a percentage. Recent studies have shown that RDW played a key role in cardiovascular disease progression through various physiological and pathological manifestations [1–3]. Increased attention has been focused on the predictive and prognostic value of RDW in patients with coronary heart disease, heart failure, atrial fibrillation, aortic valve replacement surgery, acute pulmonary embolism and cerebral infarction [4–10]. Elevated RDW is associated with a higher risk of perioperative stroke/transient ischaemic attack and death in patients undergoing heart valve surgery [11]. Moreover, high RDW is also associated with cardiovascular events and mortality in patients after myocardial infarction [12].

The Global Registry of Acute Coronary Events (GRACE) risk score has been developed to assess the prognosis of patients with acute coronary syndromes (ACS) [13]. It was calculated with admission variables, including age, heart rate, systolic blood pressure, Killip class, ST-segment deviation, serum creatinine, cardiac markers and cardiac arrest. Detection of a high GRACE risk score is associated with poor prognosis [14]. European Society of Cardiology (ESC) and American Heart Association (AHA) guidelines emphasize the important prognostic value of GRACE risk score, and recommend its use for risk evaluation routinely [15–16].

Understanding whether RDW and GRACE score play an important role in predicting the prognosis of ST-segment elevation myocardial infarction (STEMI) would assist in greater accuracy with respect to the risk stratification of STEMI patients and would also provide the opportunity for early interventions to improve patient prognosis. No previous study has evaluated the combined value of RDW and GRACE risk score in predicting the major adverse cardiac event (Mace) in STEMI patients, nor its association with Mace in the long term follow-up. Therefore, we attempt to explore the prognostic value of the two parameters separately and combinedly in predicting the cardiovascular outcomes and mortality in STEMI patients undergoing primary percutaneous coronary intervention (PCI).

## RESULTS

### Demographic variables and baseline clinical characteristics

During the follow-up period (33.5 ± 7.1 months), a total of 126 Mace were recorded, including 54 (13.8%) cases of cardiac deaths, and 72 (18.5%) cases of nonfatal myocardial infarction. There were no significant difference in clinical characteristics, including demographic variables, angiographic information and several biochemical parameters between Mace group and non-Mace group. However, Mace occurred most frequently in the patients who had a higher Killip classification, higher GRACE score level, and higher concentration of RDW and hsCRP, as compared to non-Mace patients (Table 1).

**Table 1:** Demographic variables and baseline clinical characteristics

Characteristics	MACE group *n =* 126	Non-MACE group *n =* 264	*P* value
Age, years	62.5 ± 11.5	61.5 ± 11.2	0.424
Gender, male	100 (79.4)	195 (73.9)	0.237
BMI, Kg/m^2^	23.7 ± 2.98	23.8 ± 3.58	0.844
Initial heart rate, beats/min	74.5 ± 15.0	74.2 ± 11.2	0.844
Current smoker	65 (51.6)	115 (43.6)	0.137
Hypertension	61 (48.4)	147 (55.7)	0.178
Diabetes mellitus	25 (19.8)	66 (25.0)	0.260
Family history	32 (25.4)	54 (20.5)	0.271
Medication at discharge			
Aspirin	125 (99.2)	262 (99.2)	1.000
Clopidogrel	125 (99.2)	263 (99.6)	0.542
Statins	124 (98.4)	261 (98.9)	0.660
Beta-blocker	102 (81.0)	212 (80.3)	0.880
ACEI/ARB	87 (69.0)	189 (71.6)	0.606
Diuretic	42 (33.3)	84 (31.8)	0.765
Angina history	25 (19.8)	67 (25.4)	0.228
Anterior infarction	65 (51.6)	112 (42.4)	0.089
Killip class ≥ 2	42 (33.3)	47 (17.8)	0.001
Number of diseased vessels			0.136
1-vessel disease	86	161	
2-vessel disease	33	94	
3-vessel disease	7	9	
Number of stents per patient	1.24 ± 0.58	1.28 ± 0.63	0.490
Gensini score	49.1 ± 26.9	46.5 ± 26.2	0.374
LVEDD, mm	48.5 ± 4.8	47.5 ± 6.5	0.145
LVEF, %	58.8 ± 8.3	59.9 ± 9.3	0.264
Creatinine, umol/L	71.8 ± 20.2	72.1 ± 20.4	0.915
Uric acid, mmol/L	312.0 ± 101.0	305.8 ± 96.0	0.556
Potassium, mmol/L	4.12 ± 0.52	4.19 ± 0.44	0.232
Total cholesterol, mmol/L	4.42 ± 1.03	4.33 ± 1.07	0.438
Triglyceride, mmol/L	1.64 ± 0.94	1.78 ± 1.14	0.249
HDL-C, mmol/L	1.09 ± 0.24	1.12 ± 0.28	0.429
LDL-C, mmol/L	2.57 ± 0.76	2.42 ± 0.76	0.076
HsCRP, g/L	9.61 ± 5.05	6.96 ± 4.89	< 0.001
White blood cell count	9.90 ± 3.79	9.57 ± 2.49	0.302
Hemoglobin, g/L	137.0 ± 17.3	137.6 ± 18.9	0.794
Platelet count	223.5 ± 62.0	212.4 ± 60.1	0.092
RDW, %	13.5 ± 0.99	12.9 ± 0.87	< 0.001
GRACE score	133.0 ± 31.3	112.3 ± 23.0	< 0.001

Values are presented as mean ± SD or n (%). Abbreviations: ACEI: Angiotensin-converting enzyme inhibitor, ARB: Angiotensin receptor blocker, LVEDD: Left ventricular end-diastolic dimension, LVEF: Left ventricular ejection fraction, HDL-C: High-density lipoprotein-cholesterol, LDL-C: Low-density lipoprotein-cholesterol, hsCRP: Hypersensitive C reactive protein, RDW: Red blood cell distribution width, GRACE score: The Global Registry of Acute Coronary Event risk score.

### Associations between demographic variables and RDW

**Figure 1:**
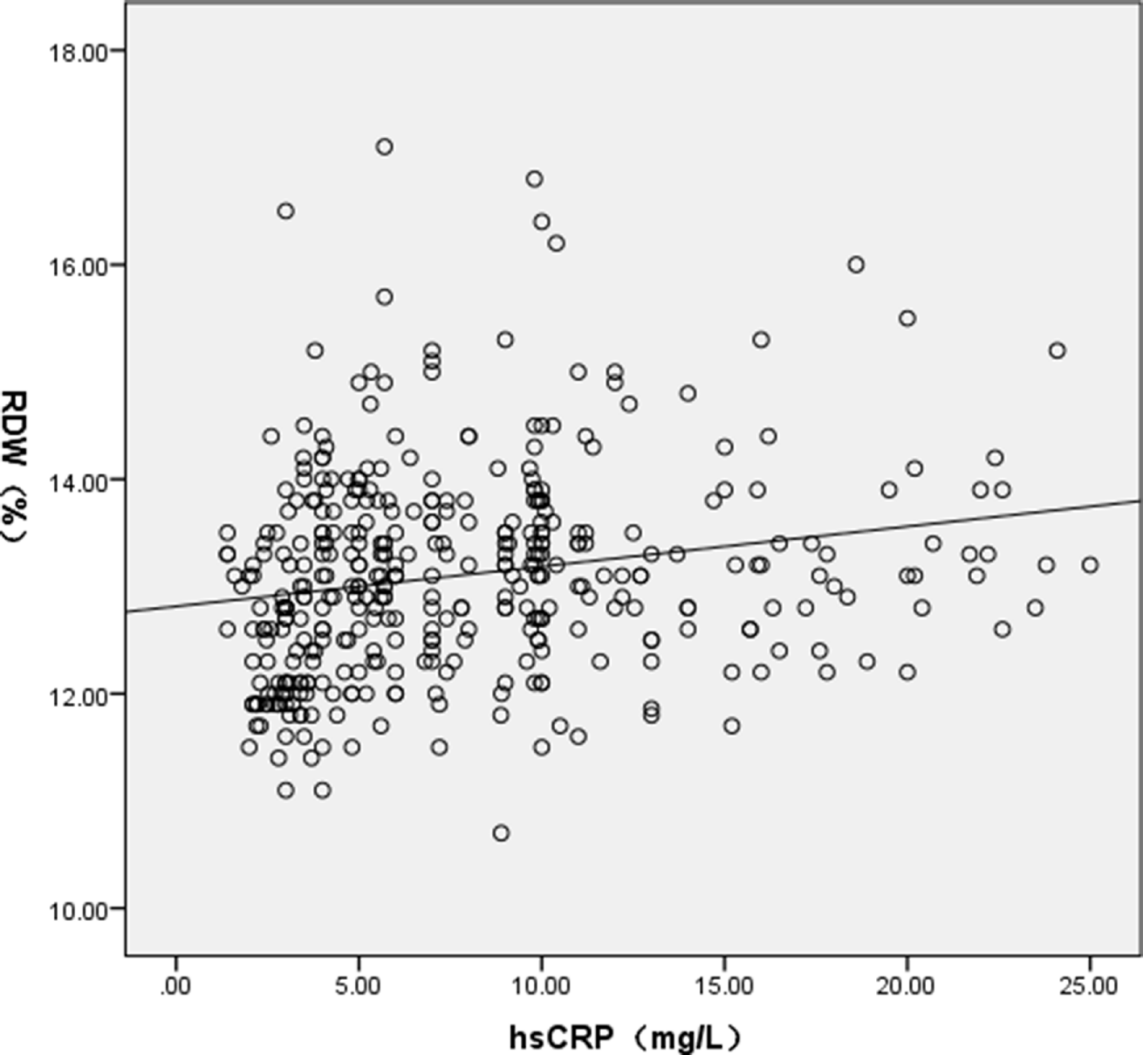
A scatter plot showing the relationship between RDW and hsCRP RDW: Red blood cell distribution width, hsCRP: Hypersensitive C reactive protein.

No significant association has been found between either RDW (r = 0.063, *P* = 0.216) or hsCRP (r = 0.007, *P* = 0.895) and GRACE score. However, a remarkable correlation was observed between hsCRP and RDW (r = 0.198, *P* < 0.001) (Figure 1).

### Cox regression analysis for Mace and cardiac death

Univariate analysis of data showed that RDW, GRACE score, hsCRP and Killip class were strongly associated with Mace. Moreover, RDW, GRACE score, hsCRP, LDL-C, and anterior infarction were significantly associated with cardiac death (Table 2). A multivariate Cox regression model showed that RDW (HR = 1.735, 95% CI 1.439–2.091, *P* < 0.001), GRACE score (HR = 1.022, 95% CI 1.017–1.028, *P* < 0.001), hsCRP (HR = 1.056, 95% CI 1.025–1.088, *P* < 0.001), current smoker (HR = 1.668, 95% CI 1.166–2.387, *P* = 0.005), anterior infarction (HR = 1.673, 95% CI 1.163–2.407, *P* = 0.006), and angina history (HR = 0.522, 95% CI 0.329–0.829, *P* = 0.006) were associated with Mace. Moreover, RDW (HR = 1.562, 95% CI 1.174–2.078, *P* = 0.002), GRACE score (HR = 1.015, 95% CI 1.007–1.023, *P* = 0.001), hsCRP (HR = 1.054, 95% CI 1.007–1.103, *P* = 0.024), LDL-C (HR = 1.499, 95% CI 1.092–2.059, *P* = 0.012), and anterior infarction (HR = 2.221, 95% CI 1.268–3.892, *P* = 0.005) were independent predictors of cardiac death (Table 2). After adjusting for age, current smoker, anterior infarction, angina history, and LDL-C, RDW (HR = 1.702, 95% CI 1.436–2.017, *P* < 0.001)/(HR = 1.546, 95% CI 1.198–1.994, *P* < 0.001) and GRACE (HR = 1.019, 95% CI 1.014–1.023, *P* < 0.001)/(HR = 1.015, 95% CI 1.008–1.023, *P* < 0.001) score were still significantly associated with Mace and cardiac death.

**Table 2:** Cox regression analysis for MACE and cardiac death

Variable	Univariate HR (95% CI)	*P*-value	Multivariate HR (95% CI)	*P*-value
**MACE**				
RDW	1.697 (1.449–1.988)	< 0.001	1.735 (1.439–2.091)	< 0.001
GRACE score	1.020 (1.015–1.025)	< 0.001	1.022 (1.017–1.028)	< 0.001
hsCRP	1.076 (1.045–1.108)	< 0.001	1.056 (1.025–1.088)	< 0.001
Current smoker	1.302 (0.918–1.846)	0.139	1.668 (1.166–2.387)	0.005
Anterior infarction	1.366 (0.963–1.937)	0.080	1.673 (1.163–2.407)	0.006
Killip class	1.610 (1.327–1.953)	< 0.001	-	-
Angina history	0.756 (0.488–1.171)	0.210	0.522 (0.329–0.829)	0.006
LDL-C	1.215 (0.970–1.522)	0.090	-	-
**Cardiac death**				
RDW	1.534 (1.208–1.948)	< 0.001	1.562 (1.174–2.078)	0.002
GRACE score	1.016 (1.008–1.024)	< 0.001	1.015 (1.007–1.023)	< 0.001
hsCRP	1.072 (1.025–1.120)	0.002	1.054 (1.007–1.103)	0.024
Killip class	1.326 (0.981–1.791)	0.066	-	-
Anterior infarction	2.184 (1.257–3.794)	0.006	2.221 (1.268–3.892)	0.005
LDL-C	1.563 (1.126–2.169)	0.008	1.499 (1.092–2.059)	0.012

Abbreviations: HR: hazard ratio, CI: confidence interval, RDW: Red blood cell distribution width, GRACE score: The Global Registry of Acute Coronary Event risk score, hsCRP: Hypersensitive C reactive protein, LDL-C: Low-density lipoprotein-cholesterol, LVEDD: Left ventricular end-diastolic dimension.

### Diagnostic value of RDW and GRACE score for Mace

**Figure 2:**
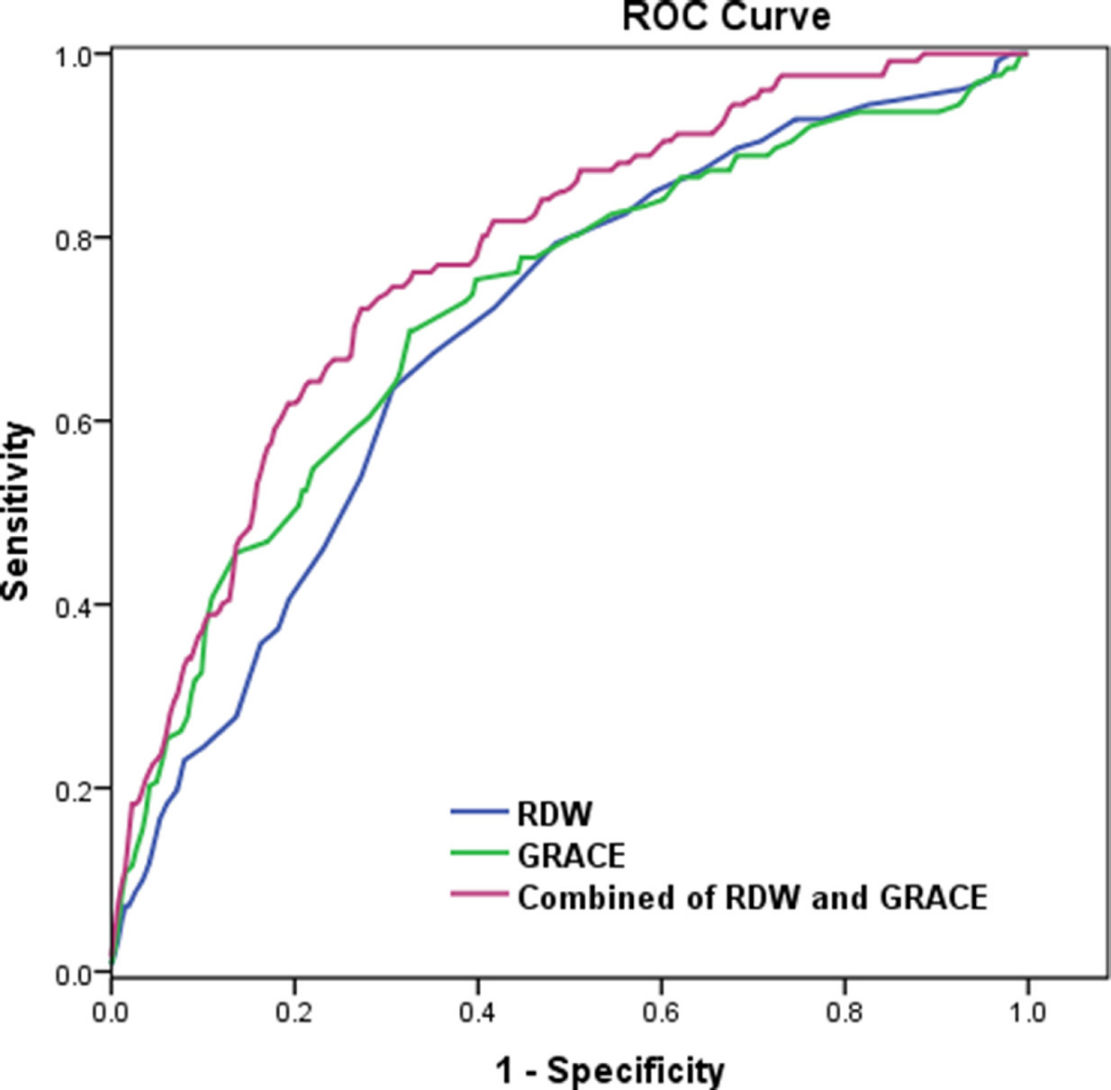
The receiver-operating characteristic (ROC) curve for red cell distribution width (RDW), GRACE score, and the combined value for predicting major adverse cardiac events (Mace) (Figure 2)

ROC analysis was conducted to determine the cut-off value of RDW and GRACE score for the prediction of the occurrence of Mace. The cutoff of RDW was 13.25%, with 63.5% sensitivity and 69.3% specificity (AUC = 0.694, 95% CI 0.639–0.750, *P* < 0.001), And the cutoff of GRACE score was 119.5, with 69.8% sensitivity and 67.4% specificity (AUC = 0.721, 95% CI 0.666–0.777, *P* < 0.001). Combining RDW and GRACE risk score yielded a much more valuable predictive value (AUC = 0.775, 95% CI: 0.727–0.824, *P* < 0.001) with sensitivity and specificity achieving 72.2% and 73.5% successively (Figure 2).

### The Kaplan–Meier survival analysis for cardiac death

**Figure 3:**
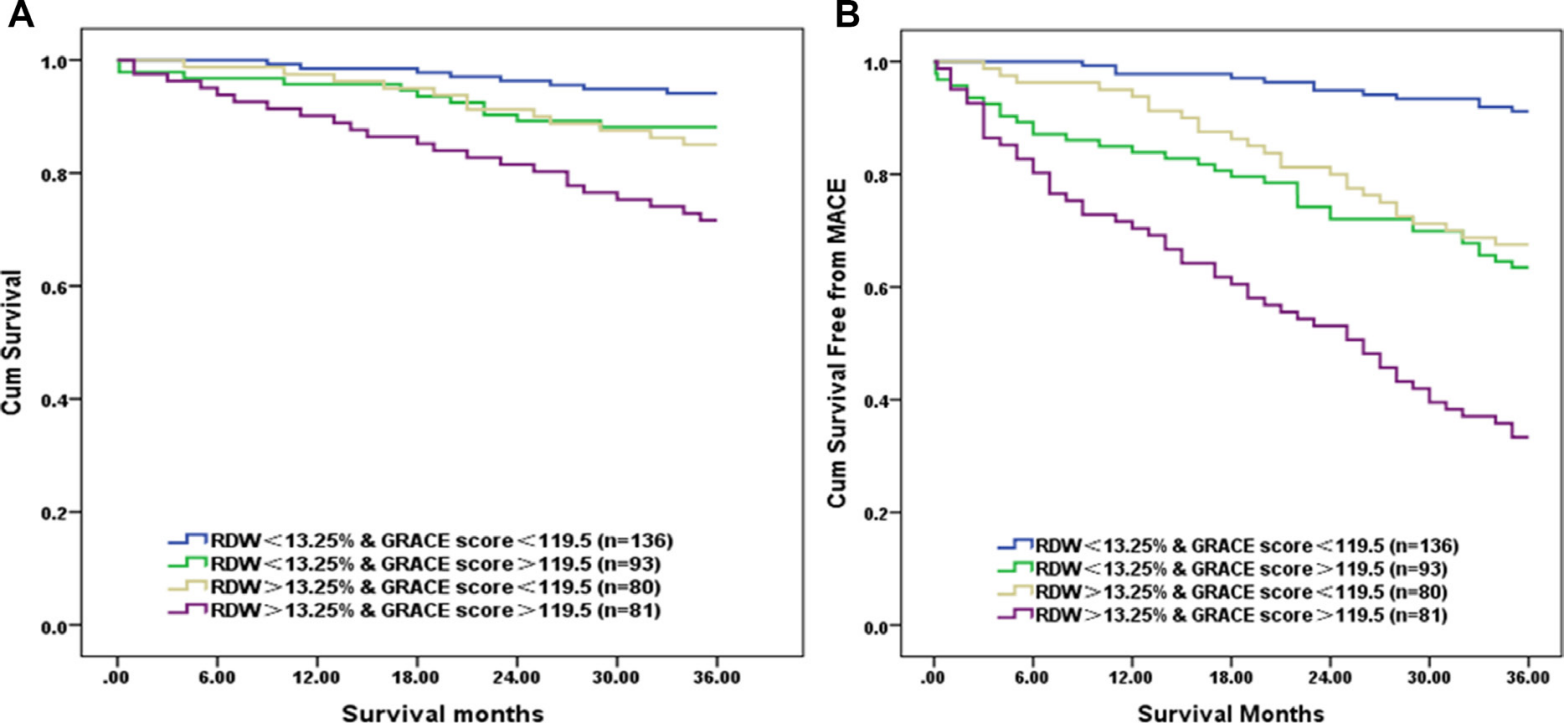
Kaplan–Meier survival analysis for cardiac death (**A**) and Mace (**B**). All patients were stratified into 4 groups based on cut-off values of RDW (13.25%) and GRACE score (119.5). The group with high RDW (> 13.25%) and high GRACE score (> 119.5) had the worse prognosis than other groups (Log-Rank χ^2^ = 24.684, 92.252, *P* < 0.001). RDW: Red blood cell distribution width, GRACE score: The Global Registry of Acute Coronary Event risk score, Mace: major adverse cardiac events.

Patients were further divided into four groups according to the cutoff value of RDW (13.25%) and GRACE score (119.5): low RDW (< 13.25%) and low GRACE score (< 119.5) group (*n* = 136), low RDW (< 13.25%) and high GRACE score (< 119.5) group (*n* = 93), high RDW (> 13.25%) and low GRACE score (< 119.5) group (*n* = 80), as well as high RDW (> 13.25%) and high GRACE score (> 119.5) group (*n* = 81). Result showed that patients with high RDW and high GRACE score had the worst outcomes. Correspondingly, Kaplan–Meier survival analysis revealed that the cumulative survival rate was the lowest in the high RDW and high GRACE score group (Log-Rank χ^2^ = 24.684, *P* < 0.001) (Figure 3A). Significant intergroup differences (*p* < 0.001) were found between high RDW and high GRACE score group and all the rest groups as well as between high RDW and low GRACE score group and high RDW and low GRACE score group (Log-Rank χ^2^ = 5.009, *P* = 0.025). Moreover, Kaplan–Meier survival analysis revealed that the cumulative survival rate free from Mace was the lowest in the high RDW and high GRACE score group (Log-Rank χ^2^ = 92.252, *P* < 0.001) (Figure 3B).

## DISCUSSION

The value of GRACE risk score in assessing prognosis and making treatment decisions has been clearly demonstrated in patients with ACS [13–14]. Current guidelines recommend its use for routine risk evaluation [15–16]. The predictive and prognostic value of RDW for adverse outcomes has also been demonstrated in patients with various diseases [4–10]. Subsequent studies have confirmed the significance of RDW level as an independent predictor for future cardiovascular events in ACS patients [17–18]. Ghaffari et al reported that an elevated baseline RDW could predict adverse outcomes in patients with STEMI undergoing thrombolytic therapy, during a follow-up period of (7.7 ± 3.2) months [19]. Isik et al studied the effects of RDW level on long-term prognosis in 96 STEMI patients who underwent primary PCI, and finally found that admission RDW level was an independent predictor of long term Mace [20]. To our knowledge, the main finding of our study is the first report showing the combined value of RDW and the GRACE score in predicting Mace at 36-month follow-up in patients with STEMI who underwent primary PCI.

The GRACE risk score models have been developed to predict risk within six months from discharge, and the models have been tested and validated in several studies and retained good predictive value for cardiovascular events up to five years in ACS patients [13, 21]. Our study demonstrated that GRACE risk score can independently predict Mace and cardiac death at 36-month follow-up in STEMI patients undergoing primary PCI. However, both our current study and several previous studies found that this scoring system still needs to be improved [22–24]. The GRACE risk score system might have some limitation as various physiological and pathological processes, such as oxidative stress and inflammation, are not wholly covered by the system. Parenica et al study showed that combinations of oxidative stress parameters including Nitrite/nitrate and superoxide dismutase with the GRACE score could enhance risk discrimination in patients with STEMI treated by primary PCI [22]. In the SPUM-ACS Biomarker Cohort study, 1892 ACS patients were enrolled at four Swiss university hospitals between 2009 and 2012. The primary composite endpoint including all-cause mortality and non-fatal recurrent myocardial infarction was achieved in all patients during 12-month follow-up. The study revealed that the inflammation biomarkers of hsCRP have an additional value to the prognostic properties of the GRACE score for the prediction of Mace [24]. Moreover, our study found that adding RDW to the GRACE risk score system could enhance the predictive power in patients with STEMI who underwent primary PCI.

The prognostic value of RDW for short-term or long-term cardiovascular events have been demonstrated in STEMI patients [20, 25]. In a meta-analysis involving 80216 patients from 22 studies, RDW was a remarkably strong predictor of all-cause mortality (HR = 1.80, 95% CI:1.35–2.41, *P* < 0.001) and non-fatal adverse events (HR = 1.86, 95% CI:1.50–2.31, *P* < 0.001), although there was significant heterogeneity in the different studies [26]. In our study, the RDW level and GRACE score of Mace group are higher than those of non-Mace group. According to Univariate and multivariate Cox regression analyses, RDW and GRACE score were found to be the independent predictors of cardiovascular events in long-term follow-up, which was consistent with the results of previous studies [20, 21]. In addition, our study also found that RDW was highly associated with the risk of Mace and cardiac death in STEMI patients who underwent primary PCI. The Kaplan–Meier analysis revealed that RDW and GRACE score can be important prognostic markers of negative long-term outcomes. Moreover, these results confirmed that RDW added discriminatory predictive value to the GRACE score. This added value was shown by the significant increase in AUC from 0.722 to 0.775 for the Mace during follow-up. Therefore, the combination of RDW and GRACE score could help evaluate long-term cardiovascular risk and could also provide an opportunity for early interventions to improve the prognosis in patients with STEMI who underwent primary PCI.

Exact physiological mechanisms between RDW and cardiovascular outcomes are not clearly understood. Several previous studies suggest that the level of RDW may reflect the extent oxidative stress, chronic subclinical inflammation, and lipid abnormalities [1, 27, 28]. Similar to previous studies [29], a significant positive correlation between hsCRP and RDW was established in our study, which indicating that RDW was significantly related to the inflammatory response intensity in STEMI patients undergoing primary PCI. Additionally, oxidative stress and inflammation result in disorders of iron metabolism and decrease the production of and response to erythropoietin, which thus might contribute to an increased RDW level [1, 27]. However, contrary to previous studies [28], our data failed to find the correlation between RDW and lipids. Neither have other authors found an association between RDW and lipids [30].

Despite that personalization therapy was recommended in high risk patients, new anti-atherosclerotic therapies were still lack in current clinical practice. Our results may have potential implications for improved prediction and development of novel therapies if confirmed in independent larger studies in future.

## MATERIALS AND METHODS

### Study population

During the period from May 2012 until May 2014, a total of 390 STEMI patients were recruited in Luoyang central hospital affiliated to Zhengzhou University. All patients undergoing primary PCI within less than 12 h after the onset of symptoms. There were 295 males and 95 females among these patients. The average age was about (55.25 ± 6.79) years. All patients received loading doses of clopidogrel (600 mg) and aspirin (300 mg) before primary PCI and admitted to the coronary care unit after the surgery. Daily clopidogrel (75 mg) and aspirin (100 mg) were continued. STEMI is a syndrome defined as a typical chest pain of ischemia in association with persistent electrocardiographic ST elevation or new onset of complete left bundle branch block, and subsequent increasing of biomarkers of myocardial necrosis. Patients were excluded from the present study if they had history of revascularization, cardiomyopathies, valvular heart disease, chronic pulmonary disease, chronic renal failure, untreated infection, thyroid disease or previous myocardial infarction.

### Study parameters

The current study is a prospective study approved by the Ethics Committee of Luoyang central hospital affiliated to Zhengzhou University. All patients underwent coronary angiography performed by the experienced interventional cardiologists who had no knowledge of the patients’ clinical information. Each coronary artery was displayed in at least two different plane images using standard Judkins technique. The degree of coronary artery stenosis was determined by diameter method. Stenosis over 50% was defined as meaningful lesions. Gensini Score was used to determine the severity of coronary lesions according to the method described previously [31]. The Killip classification was based on the physical examination and the development of heart failure in order to predict and stratify the risk of mortality. All patients were classified in the following way: Killip class I, no clinical sign of heart failure. Killip class II, rales or crackles in the lungs, an S3 and elevated jugular venous pressure. Killip class III, acute pulmonary edema. Killip class IV, systolic blood pressure lower than 90 mmHg, cardiogenic shock. The GRACE risk score was calculated by the following variables [13], including age, heart rate, systolic blood pressure, creatinine, in-hospital PCI, ST-segment depression, elevated cardiac enzyme, history of myocardial infarction, and congestive heart failure (http://www.outcomes-umassmed.org).

The baseline data was collected in all cases, including gender, age, body mass index (BMI), current smoker, family history, hypertension, diabetes mellitus, angina history, anterior infarction, initial heart rate, and medication at discharge. On admission, white blood cell count (WBC), RDW, hemoglobin (HGB), platelet count (PLT), potassium (K^+^), creatinine (Cr) and uric acid (UA) were measured before the coronary angiography. In addition, the levels of total cholesterol (TC), high-density lipoprotein-cholesterol (HDL-C), low-density lipoprotein-cholesterol (LDL-C), triglyceride (TG) and hypersensitive C reactive protein (hsCRP) were obtained from fasting blood of the morning after admission. Blood was routinely measured by Sysmex XE2100 hematology analyzer and Beckman AU680 automated chemistry analyzer in the central laboratory of our hospital. Philips IE33 color Doppler ultrasound was employed to measure left ventricular end-diastolic dimension (LVEDD) and left ventricular ejection fraction (LVEF).

### Clinical outcomes and data collection

All patients are routinely followed up for 36 months since their first admission. Clinical follow-up was carried out through patient visits. We reviewed the medical records if patient had readmitted to hospital. To minimize the loss to follow-up, we interviewed patients or their close relatives by telephone. Ultimately, Follow-up was 100% complete. The primary endpoints were defined as major adverse cardiac event (Mace), including cardiac death and nonfatal myocardial infarction (typical chest pain of ischemia in association with ST segment deviation, and subsequent increasing of biomarkers of myocardial necrosis). Sudden unexpected death was classified as cardiac death when it occurred outside the hospital.

### Statistical analysis

All analyses were performed using Software IBM SPSS statistics for windows version 23 (IBM Corp., Armonk, NY). Continuous variables were expressed as mean ± standard deviation (SD). The normality of data was examined. If data conformed to normality, group comparisons would be performed by independent samples *t* test. If not, nonparametric test would be employed. Categorical variables were expressed as percentages and were compared by *χ2* test. Associations between continuous variables were assessed by Pearson test. Univariate and multivariate Cox regression analyses were used to identify the relative risks (hazard ratio [HR]) for death and Mace associated with clinical risk factors, demographic and angiographic variables, such as gender, age, BMI, current smoker, angina history, hypertension, diabetes mellitus, family history, anterior infarction, Killip class, medication at discharge, HGB, PLT, total cholesterol, triglyceride, HDL-C, LDL-C, potassium, creatinine, uric acid, LVEDD, LVEF, Gensini score, number of diseased vessels and stents per patient, as well as with GRACE score, hsCRP and RDW levels. Receiver operating characteristic (ROC) curves was to ensure the best possible risk categorization. We also generated ROC curves to determine the sensitivities and specificities of hsCRP, GRACE score and RDW. Then, based on the cut-off values, patient outcomes were assessed using Kaplan–Meier survival curves with Log-rank test. A two-tailed, *P* value < 0.05 was regarded as statistically significant.

### Limitations

The current study has several potential limitations. Firstly, without wholly understanding of the detailed mechanisms linking the increased cardiovascular events in patients to higher levels of RDW, it seems to be too early to name RDW as a prognostic predictor in STEMI patients undergoing primary PCI. Secondly, it was a single-center real-world study that included a relatively small number of Chinese individuals. Multi-center studies may provide different insight. Thirdly, the cut-off value of RDW in the current study is 13.2%. Several previous studies have also confirmed the significance of RDW level as an independent predictor for future cardiovascular events in ACS patients but the cut-off values are not the same [32, 33], making assess comparability between studies more complex. It might attribute to different diseases the patients suffering from, as ACS includes unstable angina, STEMI as well as non ST-segment elevation myocardial infarction (USTEMI). Additionally, different genetic background of the participants might also influence the level of RDW. Another potential reason is the type of hematology analyzer used. Blood in the current study was measured by Sysmex XE2100 hematology analyzer and Beckman AU680 automated chemistry analyzer in the central laboratory of our hospital. Because each device has a unique calibration and therefore it could affect the comparability of results.

## CONCLUSIONS

The present study showed that combination of RDW level and GRACE score may be valuable and simple independent predictor for Mace and cardiac death in patients with STEMI who underwent primary PCI. They may be useful tools for risk stratification and may indicate long-term clinical outcomes. Our results may have potential implications for improved prediction and development of novel therapies. More studies with larger-scale population are needed to validate the prognostic significance.
